# The Anticancer Properties of Cordycepin and Their Underlying Mechanisms

**DOI:** 10.3390/ijms19103027

**Published:** 2018-10-04

**Authors:** So Young Yoon, Soo Jung Park, Yoon Jung Park

**Affiliations:** 1 Department of Nutritional Science and Food Management, Ewha Womans University, Seoul 03760, Korea; syyoon0504@gmail.com; 2 Department of Sasang Constitutional Medicine, Woosuk University, Jeollabuk-do 55338, Korea; soojungpark@woosuk.ac.kr

**Keywords:** cordycepin, *Cordyceps*, anticancer, adenosine receptors, death receptors

## Abstract

*Cordyceps* is a genus of ascomycete fungi that has been used for traditional herbal remedies. It contains various bioactive ingredients including cordycepin. Cordycepin, also known as 3-deoxyadenosine, is a major compound and has been suggested to have anticancer potential. The treatment of various cancer cells with cordycepin in effectively induces cell death and retards their cancerous properties. However, the underlying mechanism is not fully understood. Recent evidence has shed light on the molecular pathways involving cysteine-aspartic proteases (caspases), mitogen-activated protein kinases (MAPKs), and glycogen synthase kinase 3 beta (GSK-3β). Furthermore, the pathways are mediated by putative receptors, such as adenosine receptors (ADORAs), death receptors (DRs), and the epidermal growth factor receptor (EGFR). This review provides the molecular mechanisms by which cordycepin functions as a singular or combinational anticancer therapeutic agent.

## 1. Introduction

Cancer is the uncontrolled growth of abnormal cells with the potential to metastasize. The treatments given to cancer patients are designed to either directly remove the cancer cells or exert a controlled death of the cancer cells by anti-proliferative and cytotoxic chemicals via cell cycle arrest and apoptotic induction. Systemic therapy using antitumor drugs, besides surgical and radiation therapies, is one of the primary approaches against cancer. The disadvantages of systemic treatment include side-effects on normal tissues and the acquisition of drug resistance, underscoring the importance of combinational treatments [1]. Recently, complementary therapies have been under much study to evaluate their potential benefits in cancer therapies as a part of combinational treatments. The treatments are now provided in clinics internationally, with the use of complementary medicine no longer limited to uneducated, underground practitioners [2]. Herbal remedies are a large part of complementary therapies. Among various herbal compounds, *Cordyceps* is emerging as one of the herbals in cancer therapies due to its anticancer properties. *Cordyceps* contains cordycepin as a major component. Cordycepin is one of the cytotoxic nucleoside analogues that were first tried as chemotherapeutic agents [3,4]. This review focuses on cordycepin as a potential anticancer drug that has complementary therapeutic activities in pro-apoptosis, anti-proliferation, and anti-metastasis in cancer cells.

## 2. *Cordyceps*: A Medicinal Herb

*Cordyceps* is a genus of parasitic fungi that parasitizes on the larvae of arthropods for its reproductive and survival purposes. The fungus was first recorded as Ben-Cao-Bei-Yao in 1694 and is usually called ‘Dong-Chong-Xia-Cao’ in China. This fungus has been used as a health supplement in Asian countries for over 300 years for suboptimal health status patients [5]. *Cordyceps* has approximately 400 species that are distributed in places where the climate is humid and subtropical. To date, *Cordyceps sinensis* and *Cordyceps militaris* have been the most broadly researched species of the fungus. Both *Cordyceps* species contain similar bioactive ingredients, including cordycepin as a major component and others such as adenosine, cordycepic acid, erogosterol, and D-mannitol. Recent studies have reported that chemical constituents extracted from *Cordyceps*, especially cordycepin, exhibit potential therapeutic characteristics for cancer [5,6,7,8]. Although the extraction yield from *Cordyceps* varies depending on the volume ratio of solvent to sample, extraction time, and ethanol concentration, in that order, the peak extraction yield of cordycepin and its derivative cordycepic acid has been reported as about 7 mg/g from *Cordyceps militaris* and 7–29% of extracted components from *Cordyceps sinensis* [5,9,10].

## 3. An Active Constituent of *Cordyceps*, Cordycepin, as an Anticancer Agent

Cordycepin, also known as 3-deoxyadenosine, has a similar structure to adenosine but lacks the 3′-hydroxyl group of the ribose moiety [3]. Adenosine is a signaling molecule that affects various types of cells, tissues, and organ systems, via both intracellular and extracellular signaling pathways [11]. It exerts its effects through cell surface receptors such as adenosine receptors (ADORAs). Cordycepin is an adenosine derivative that demonstrates multiple physiological functions, such as anti-oxidative activity, immune system activation [12], sexual performance enhancement [13], anticancer effects [14], and anti-metastatic effects [13]. 

The broad putative pharmacological actions of cordycepin have attracted a great attention as a new therapeutic for chronic diseases including diabetes and dyslipidemia [15] in recent decades. In particular, cordycepin has been suggested to have anticancer potential (Table 1) due to its structural similarity to adenosine, as the overexpression of adenosine generating enzymes and ADORAs has been correlated with tumor progression [16]. For example, it has been shown to induce apoptotic effects in breast, liver, and leukemia cancer cells by increasing caspase-3 and caspase-9, as listed in Table 1. In addition to apoptotic induction, effects on anti-proliferation and cell cycle arrest were also shown in lung cancer and glioblastoma. 

Cordycepin has been considered as a nucleoside analogue [4], which is able to be involved in the actions of DNA and/or RNA polymerases [17]. After entering into the cell, it becomes mono-, di-, and tri-phosphates of 3-deoxyadenosine [18]. Due to the structural similarity with adenosine monophosphate, it is possible that cordycepin monophosphate provokes termination of elongation by incorporating into the site where nucleic acids are supposed to incorporate [19]. This phenomenon has been shown in yeast [19,20] and mammals [21]. Furthermore, cordycepin monophosphate might inhibit the activity of phosphoribosyl-pyrophosphate amidotransferase, leading to hindered de novo purine synthesis [22].

**Figure 1:**
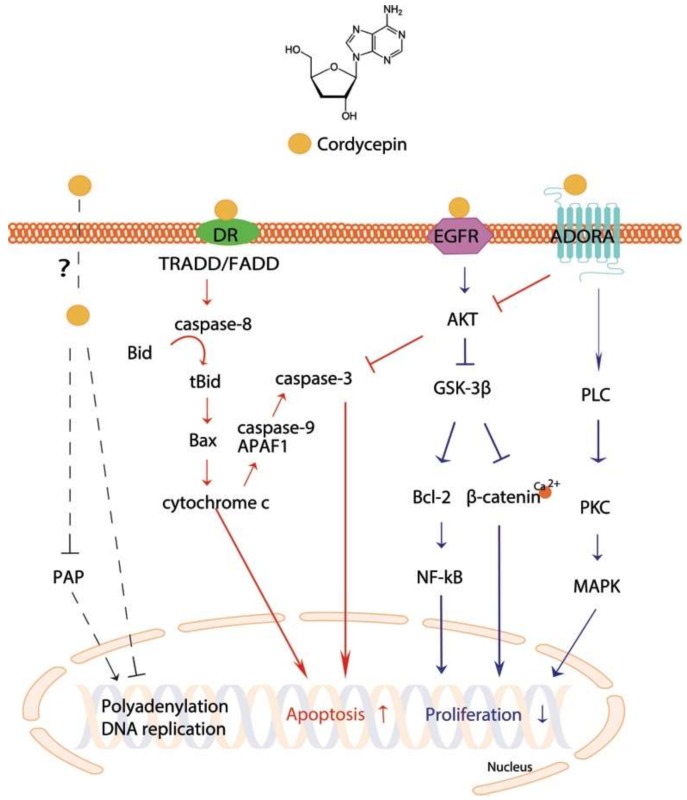
Cellular apoptotic and proliferative pathways stimulated by cordycepin. Arrows and bar-headed lines represent signaling activation and inhibition, respectively. Red- and blue-colored arrows indicate the processes involved in cell apoptosis and proliferation, respectively. Black dotted lines show processes involved in DNA and/or RNA polymerases activity.

Recently, it was shown that the apoptotic effect of cordycepin was related to dysregulated polyadenylation [23]. Polyadenylation is critical for mRNA stability and translation initiation [24], and is created by cleaving pre-mRNA at the poly(A) site and adding a poly(A) tail to the 3′ end of the cleavage product [23]. Cordycepin reduces poly(A) tails as a polyadenylation inhibitor [25] of sensitive mRNAs, such as Cdkn1a mRNA, which is a key player in cell apoptosis [26]. The polyadenylate polymerase (PAP), which is located in both the nucleus and the cytoplasm, is one of the most studied polyadenylation protein complexes [23]. In human leukemia cells, cordycepin reduced PAP activity (Figure 1) upon cordycepin treatment in a cell type-specific manner [23]. This phenomenon may be related to a reduction in polyadenylation, in particular that of apoptotic genes. However, it remains unclear how cordycepin penetrates cell membranes.

The major anticancer activity of cordycepin is likely to be mediated by cell surface receptors. Three receptors have been reported as candidates: ADORAs, death receptors (DRs), and the epidermal growth factor receptor (EGFR) (Table 2). Cordycepin has been suggested to mediate apoptotic signaling via ADORAs in glioma, melanoma, and lung carcinoma cell lines [27,28,29,30], and DRs in colon and prostate cancer cell lines [31,32]. In addition, when converted to a 5′-triphosphate-bound form [22] in vivo, it may compete with ATP in a signaling pathway [31]. It is known that ATP stimulates EGFR by a direct binding. On the contrary, 5′-triphosphate-cordycepin may fit into the binding site, possibly blocking the phosphorylation of EGFR [33]. However, to our knowledge, there is no evidence showing 5′-triphosphate-cordycepin directly binds to any of the candidate receptors. The molecular mechanisms underlying its anticancer effects are not fully understood. However, recent studies have uncovered the putative mechanisms that induce apoptosis and/or block cell proliferation as summarized below. 

## 4. Major Mechanisms Underlying the Antitumor Effects of Cordycepin

### 4.1. Caspase-Mediated Apoptotic Induction

One of the major anticancer functions is achieved by apoptotic induction. Apoptosis is a process of programmed cell death [51]. It is stimulated by intrinsic stress, such as reactive oxygen species and endoplasmic reticulum stress, in a mitochondria-dependent manner or by extrinsic signaling in a death receptor-dependent manner [31]. Death domain (DD) proteins have been reported to be central in the receptor-dependent apoptotic pathway of cancer cells. Fas-associated protein with death domain (FADD) and tumor necrosis factor receptor type 1-associated death domain protein (TRADD) are adaptor proteins that mediate the signals from the binding of extrinsic stimuli to DRs. Upon recruitment to DRs, pro-caspase-8 is cleaved and is activated to initiate a caspase cascade, by cleaving caspase-3, in human cancer cells [56]. Cordycepin has been shown to be involved in DR-mediated pathways [31,32] (Figure 1). Cordycepin treatment elevated DR3 expression in the human colon cancer cell line HT-29 [31] and DR5 expression along with Fas and BAX levels in human prostate carcinoma [32], leading to induction of cell death [31,32]. Moreover, Cordycepin treatment of NB-4 cells released cytochrome c from the mitochondria into the cytosol, triggering intrinsic apoptosis [51]. Mitochondrial translocation levels of the cell death inhibitor BAX increase and cause the release of cytochrome c in response to induction by tBid, one of the proapoptotic proteins [45]. Although anti-apoptotic Bcl-2 proteins act as gatekeepers of cytochrome c release, Bcl-2 family proteins are able to bind to apoptotic protease activating factor 1 (APAF1) molecules, facilitating the formation of an apoptosome, and the apoptosome subsequently cleaves procaspase-9 [51,57]. The effect of cordycepin on NB-4 cells, which led to release of mitochondrial cytochrome c to the cytosol, resulted in activation of caspase-9, indicating induction of apoptosis [51]. The results showed that cordycepin induces both caspase-mediated intrinsic regulation and extrinsic apoptotic regulation. These effects were confirmed in various cancer types, including liver [34], lung [43], breast [33,45,46], prostate [39,43], leukemia [3,49], and neuroblastoma [53,54] cancer cells.

### 4.2. MAPK-Mediated Apoptotic Induction and Anti-Proliferation

The ADORAs, a class of purinergic G protein-coupled receptors with adenosine as an endogenous ligand, are increasingly recognized as a promising therapeutic targets for cancer [58]. They are classified into four subtypes in humans: ADORA1, ADORA2A, ADORA2B, and ADORA3 [59,60]. In particular, ADORA2A and ADORA3 are considered as possible receptors of cordycepin [28,29,30] (Figure 1). Over expression of ADORA3 in human bladder cancer cells had similar effects to treatment with *Cordyceps* extract, whereas depletion of ADORA3 abrogated the effect of *Cordyceps* extract containing cordycepin as a major component [28]. Cordycepin treatment of C6 glioma cells also showed that cordycepin induced apoptosis via the ADORA2A pathway [27]. In turn, ADORAs can regulate the activity of PLC through a G protein, as shown in rat basophilic leukemia cells [61,62]. Thus, the results suggested that cordycepin works on cancer cells through the ADORA-G-protein-PLC pathway to induce cell apoptosis [11].

Phospholipase C (PLC) is significant in transmembrane signaling [63]. It cleaves the phospholipid phosphatidylinositol 4,5-biphosphate (PIP2) into inositol 1,4,5-triphosphate (IP3) and diacylglyceride (DAG), which, together with Ca^2+^, stimulate protein kinase C (PKC) [64]. Cordycepin elevated intracellular PLC/PKC and MAPK pathways in MA-10 mouse Leydig tumor cells to induce cell death [12]. The MAPK pathway is critical for regulation of cell survival [38] and involves various MAPK families such as extracellular signal-regulated kinases (ERKs), c-Jun N-terminal kinases (JNKs), and p38 MAP kinases [38,65]. In general, activation of ERKs stimulates cell proliferation, whereas activation of JNKs promotes cell apoptosis [40]. Several studies showed that cordycepin induced apoptosis and decreased proliferation by regulating ERK/JNK signaling or activating p38 MAPK in human bladder [38], renal [39,40], lung [41,42], leukemia [51], and glioblastoma [52] cancer cells. Joo et al. [41] reported that caveolin-1 (CAV1)-mediated phosphorylation of JNK and dephosphorylation of forkhead transcription factor 3a (Foxo3a) were observed in A549 cell apoptosis after cordycepin treatment.

Cordycepin also leads to cell cycle arrest by regulation of a MAPK-mediated pathway [38,51]. It induced G2/M cell-cycle arrest by activating JNK in human bladder cancer cells [38]. The effect of cordycepin was attenuated by SP6001259, a JNK-specific inhibitor, indicating that it was mediated by JNK. Activation of JNK upregulated expression of P21, resulting in cell cycle arrest [38]. Cordycepin not only caused G2/M-phage arrest, but also caused S-phage arrest in leukemia cells [51] and G1-phage arrest in neuroblastoma and melanoma cells [54].

### 4.3. Anti-Proliferation Effect via GSK-3β Pathway

Cordycepin treatment inhibited nuclear translocation of β-catenin in human leukemia cells by reducing β-catenin stability by inducing proteasome-dependent degradation [50]. Intriguingly, adenosine treatment had no effect on β-catenin. The cordycepin-specific suppression of β-catenin was mediated by a reduction in the phosphorylation of GSK-3β and its upstream Akt (Figure 1). Akt is known to be stimulated by EGFR and phosphoinositide-3 kinase (PI3K) in cancer [66]. In human lung cancer cells, cordycepin treatment reduced the phosphorylation level of EGFR and Akt [47]. Phosphorylation of GSK-3β is also known to be regulated by ADORA3-activated G protein signaling [67]. ADORA3-mediated G protein, which is possibly stimulated by cordycepin, tempers cyclic AMP (cAMP) formation, resulting in the suppression of protein kinase A (PKA) and subsequently GSK-3β [67]. Mitigation of GSK-3β in cancer cells leads to suppression of cyclin D1 and c-myc, which inhibits cell proliferation [67].

### 4.4. Anti-Metastatic Effect Induced by Cordycepin

It is important to acknowledge that cordycepin not only exhibits proapoptotic and anti-proliferative effects in cancer cells, but also inhibits cell metastasis in tumor cells, demonstrating its potential as a therapeutic agent in the larger scope of cancer [68]. Upon cordycepin treatment, at least in vitro, inhibition of platelet aggregation and reduction in the invasiveness of melanoma cancer cells, by inhibition of the activity of matrix metalloproteinase (MMP)-2 and MMP-9 and stimulation of tissue inhibitor of metalloproteinase (TIMP)-1 and TIMP-2, were shown [13]. Similar effects on the inhibition of cell invasion by decreased MMP-9 activity were also observed in human lung cancer cells [43] and breast cancer cells [48].

## 5. Conclusions

Despite the fact that remarkable advances in cancer treatments have improved the five-year survival rates for primary tumors, those of tumor metastasis remain below thirty-percent, emphasizing the need for advanced therapeutic approaches in cancer management [69]. Therefore, it is important to investigate combinational therapeutic strategies for cancer treatment by inhibiting proliferation and inducing apoptosis, and by preventing or overcoming metastasis. According to previous studies, cordycepin is expected to be a potential combinational therapeutic drug for cancer. Further studies are needed to fully discover the underlying mechanisms of cordycepin-mediated signaling pathways.

## Figures and Tables

**Table 1:** Current evidence for the effects of cordycepin on various tumor types and possible mechanisms.

Tumor Type	Effects	Major Mediating Signaling Pathways	Results	References
Liver cancer	Apoptosis induction	Cysteine-aspartic proteases (caspase)	- Increased caspase-3, caspase-8, caspase-9, FADD, and Bid - Decreased Bcl-2	[34]
	Apoptosis induction, Anti-proliferation	Phospholipase C (PLC)	- Increased PARP cleavage	[35,36]
Colon cancer	Cell cycle arrest	c-Jun N-terminal kinases (JNK)	- G2/M-phase cell-cycle arrest - Increased JNK and p21WAF-1 expression	[37]
Bladder cancer	Cell cycle arrest	c-Jun N-terminal kinases (JNK)	- G2/M cell-cycle arrest - Increased cJNK	[38]
Renal cancer	Apoptosis induction	ERK/JNK	- Increased JNK and caspase-3 - Decreased ERK	[39,40]
Lung cancer	Apoptosis induction	ERK/JNK	- Increased CAV1, JNK/Foxo3a, BAX, caspase-3 cleavage, and GSK-3β - Decreased ERK	[41,42]
	Apoptosis induction, Anti-proliferation, Anti-metastasis	Caspase	- Increased MMP-9 and caspase-3 - Decreased Bcl-2	[43]
	Apoptosis induction, Anti-proliferation	NF-kB	- Increased BAX, caspase-3 cleavage - Decreased NF-kB, Bcl-2, and p65 nuclear translocation	[44]
Breast cancer	Apoptosis induction	Caspase	- Increased caspase-3, caspase-9, mitochondrial translocation of BAX, and cytochrome c	[45,46,47]
	Anti-metastasis	MAPK	- Decreased Bcl-2	[48]
Prostate cancer	Apoptosis induction	Caspase	- Increased JNK and caspase-3 - Decreased ERK	[39,40]
Leukemia	Apoptosis induction	Caspase	- Increased caspase-3, caspase-8, and PARP cleavage - Decreased Bcl-2	[3,49]
	Anti-proliferation	GSK-3𝛽	- Decreased Akt, and β-catenine	[50]
	Apoptosis induction, Cell cycle arrest	MAPK	- S-phase cell-cycle arrest - Increased cytochrome c released, and caspase-9 - Decreased MAPK, p-ERK1/2, and CDK2 expression	[51]
Glioblastoma	Apoptosis induction Anti-proliferation	MAPK	- Increased p38 MAPK, caspase-3,8, PARP cleavage - Decreased Akt and Bcl-2	[52]
Neuroblastoma	Apoptosis induction Cell cycle arrest	Caspase	- sub-G1 phase cell-cycle arrest - Increased caspase-3 and PARP cleavage	[53,54]

**Table 2:** Putative receptors stimulated by cordycepin and their downstream targets.

Receptors		Downstream Targets	References
Adenosine receptors (ADORAs)	ADORA2A	Induced apoptosis via ADORA2A receptor-p53-caspase-7-PARP pathway in C6 glioma cells.	[27]
	ADORA3	Inhibition of the tumor growth was antagonized by a selective adenosine A3 receptor agonist, MRS1191 ^1^, in mouse melanoma and lung carcinoma cells via the radioligand binding assay. The involvement of adenosine A3 receptors was confirmed with the use of MRS1523 ^2^ and MRS1220 ^3^.	[28,29,30]
Death receptors (DRs)	DR3	Induced apoptosis of the human colon cancer cell HT-29 via the elevation of DR3, caspase-8, caspase-1, cleaved caspase-3 and cleaved PARP expression.	[31]
	DR5	Caused an elevation of the levels of Fas, death receptor 5 (DR5), proapoptotic BAX, and decreased anti-apoptotic Bcl-2 level.	[32]
Epidermal growth factor receptor (EGFR)		Inhibited cell proliferation and induced apoptosis via the EGFR signaling pathway in the human lung cancer cell H1975. The caspase-3 and BAX protein levels were increased, while phosphorylated expression levels of EGFR, AKT, and ERK1/2 were decreased.	[47,55]

^1^ T3-ethyl 5-benzyl 2-methyl-6-phenyl-4-phenylethynyl-1,4-(±)-dihydropyridine-3,5-dicarboxylate; ^2^ 3-Propyl-6-ethyl-5-[(ethylthio)carbonyl]-2 phenyl-4-propyl-3-pyridine carboxylate; ^3^ N-[9-Chloro-2-(2-furanyl)[1,2,4]-triazolo[1,5-c]quinazolin-5-yl]benzene acetamide.

## Abbreviations

ADORA	Adenosine receptor
APAF1	Apoptotic protease activating factor 1
Caspases	Cysteine-aspartic proteases
cAMP	Cyclic AMP
CAV-1	Caveolin-1
DAG	Diacylglyceride
DD	Death domain
DR	Death receptor
EGFR	Epidermal growth factor receptor
ERK	Extracellular signal-regulated kinase
FADD	Fas-associated protein with death domain
Foxo3a	Forkhead transcription factor
GSK-3β	Glycogen synthase kinase 3 beta
IP3	Inositol trisphosphate
JNK	c-Jun N-terminal kinase
MAPK	Mitogen-activated protein kinase
MMP	Matrix metalloproteinase
PAP	Polyadenylate polymerase
PIP2	Phosphatidylinositol 4,5-biphosphate
PI3K	Phosphoinositide-3 kinase
PKA	Protein kinase A
PKC	Protein kinase C
PLC	Phospholipase C
TIMP	Tissue inhibitor of metalloproteinase
TRADD	Tumor necrosis factor receptor type 1-associated death domain protein

## References

[1] Thomadaki H., Tsiapalis C.M., Scorilas A. (2005). Polyadenylate polymerase modulations in human epithelioid cervix and breast cancer cell lines, treated with etoposide or cordycepin, follow cell cycle rather than apoptosis induction. Biol. Chem..

[2] Cassileth B.R. (1996). Alternative and complementary cancer treatments. Oncologist.

[3] Jeong J.-W., Jin C.-Y., Park C., Hong S.H., Kim G.-Y., Jeong Y.K., Lee J.-D., Yoo Y.H., Choi Y.H. (2011). Induction of apoptosis by cordycepin via reactive oxygen species generation in human leukemia cells. Toxicol. In Vitro.

[4] Tuli H.S., Sharma A.K., Sandhu S.S., Kashyap D. (2013). Cordycepin: A bioactive metabolite with therapeutic potential. Life Sci..

[5] Yue K., Ye M., Zhou Z., Sun W., Lin X. (2013). The genus Cordyceps: A chemical and pharmacological review. J. Pharm. Pharmacol..

[6] Andersen S.B., Gerritsma S., Yusah K.M., Mayntz D., Hywel-Jones N.L., Billen J., Boomsma J.J., Hughes D.P. (2009). The life of a dead ant: The expression of an adaptive extended phenotype. Am. Nat..

[7] Tian X., Li Y., Shen Y., Li Q., Wang Q., Feng L. (2015). Apoptosis and inhibition of proliferation of cancer cells induced by cordycepin. Oncol. Lett..

[8] Chen X., Wang Y., Liu J., Xu P., Zhang X.M., Tian Y.Y., Xue Y.M., Gao X.Y., Liu Y., Wang J.H. (2015). Synergistic effect of HMGB1 knockdown and cordycepin in the K562 human chronic myeloid leukemia cell line. Mol. Med. Rep..

[9] Song J.F., Liu C.Q., Li D.J., Jin B.Q. (2007). Optimization of cordycepin extraction from cultured *Cordyceps militaris* by HPLC-DAD coupled with uniform design. J. Chem. Technol. Biotechnol..

[10] Wang H.-J., Pan M.-C., Chang C.-K., Chang S.-W., Hsieh C.-W. (2014). Optimization of ultrasonic-assisted extraction of cordycepin from *Cordyceps militaris* using orthogonal experimental design. Molecules.

[11] Sheth S., Brito R., Mukherjea D., Rybak L.P., Ramkumar V. (2014). Adenosine receptors: Expression, function and regulation. Int. J. Mol. Sci..

[12] Pao H.-Y., Pan B.-S., Leu S.-F., Huang B.-M. (2012). Cordycepin stimulated steroidogenesis in MA-10 mouse Leydig tumor cells through the protein kinase C pathway. J. Agric. Food Chem..

[13] Nakamura K., Shinozuka K., Yoshikawa N. (2015). Anticancer and antimetastatic effects of cordycepin, an active component of *Cordyceps sinensis*. J. Pharm. Sci..

[14] Yao W.-L., Ko B.-S., Liu T.-A., Liang S.-M., Liu C.-C., Lu Y.-J., Tzean S.-S., Shen T.-L., Liou J.-Y. (2014). Cordycepin suppresses integrin/FAK signaling and epithelial-mesenchymal transition in hepatocellular carcinoma. Anti-Cancer Agents Med. Chem..

[15] Li X., Zhou Y., Zhang X., Cao X., Wu C., Guo P. (2017). Cordycepin stimulates autophagy in macrophages and prevents atherosclerotic plaque formation in ApoE-/-mice. Oncotarget.

[16] Kazemi M.H., Raoofi Mohseni S., Hojjat-Farsangi M., Anvari E., Ghalamfarsa G., Mohammadi H., Jadidi-Niaragh F. (2018). Adenosine and adenosine receptors in the immunopathogenesis and treatment of cancer. J. Cell. Physiol..

[17] Kuchta R.D. (2010). Nucleotide Analogues as Probes for DNA and RNA Polymerases. Curr. Protocols Chem. Biol..

[18] Klenow H. (1963). Formation of the mono-, di-and triphosphate of cordycepin in Ehrlich ascites-tumor cells in vitro. Biochim. Biophys. Acta.

[19] Holbein S., Wengi A., Decourty L., Freimoser F.M., Jacquier A., Dichtl B. (2009). Cordycepin interferes with 3′ end formation in yeast independently of its potential to terminate RNA chain elongation. RNA.

[20] Horowitz B., Goldfinger B.A., Marmur J. (1976). Effect of cordycepin triphosphate on the nuclear DNA-dependent RNA polymerases and poly (A) polymerase from the yeast, *Saccharomyces cerevisiae*. Arch. Biochem. Biophys..

[21] Müller W.E., Seibert G., Beyer R., Breter H.J., Maidhof A., Zahn R.K. (1977). Effect of cordycepin on nucleic acid metabolism in L5178Y cells and on nucleic acid-synthesizing enzyme systems. Cancer Res..

[22] Rottman F., Guarino A.J. (1964). The inhibition of phosphoribosyl-pyrophosphate amidotransferase activity by cordycepin monophosphate. Biochim. Biophys. Acta.

[23] Thomadaki H., Scorilas A., Tsiapalis C.M., Havredaki M. (2008). The role of cordycepin in cancer treatment via induction or inhibition of apoptosis: Implication of polyadenylation in a cell type specific manner. Cancer Chemother. Pharmacol..

[24] Wahle E., Rüegsegger U. (1999). 3′-End processing of pre-mRNA in eukaryotes. FEMS Microbiol. Rev..

[25] Imesch P., Goerens A., Fink D., Fedier A. (2012). MLH1-deficient HCT116 colon tumor cells exhibit resistance to the cytostatic and cytotoxic effect of the poly (A) polymerase inhibitor cordycepin (3’-deoxyadenosine) in vitro. Oncol. Lett..

[26] Wong Y.Y., Moon A., Duffin R., Barthet-Barateig A., Meijer H.A., Clemens M.J., de Moor C.H. (2010). Cordycepin inhibits protein synthesis and cell adhesion through effects on signal transduction. J. Biol. Chem..

[27] Chen Y., Yang S.-H., Hueng D.-Y., Syu J.-P., Liao C.-C., Wu Y.-C. (2014). Cordycepin induces apoptosis of C6 glioma cells through the adenosine 2A receptor-p53-caspase-7-PARP pathway. Chem.-Biol. Interact..

[28] Cao H.-L., Liu Z.-J., Chang Z. (2017). Cordycepin induces apoptosis in human bladder cancer cells via activation of A3 adenosine receptors. Tumor Biol..

[29] Nakamura K., Yoshikawa N., Yamaguchi Y., Kagota S., Shinozuka K., Kunitomo M. (2006). Antitumor effect of cordycepin (3′-deoxyadenosine) on mouse melanoma and lung carcinoma cells involves adenosine A3 receptor stimulation. Anticancer Res..

[30] Yoshikawa N., Yamada S., Takeuchi C., Kagota S., Shinozuka K., Kunitomo M., Nakamura K. (2008). Cordycepin (3′-deoxyadenosine) inhibits the growth of B16-BL6 mouse melanoma cells through the stimulation of adenosine A 3 receptor followed by glycogen synthase kinase-3β activation and cyclin D 1 suppression. Naunyn-Schmiedeberg’s Arch. Pharmacol..

[31] Lee S.Y., Debnath T., Kim S.-K., Lim B.O. (2013). Anti-cancer effect and apoptosis induction of cordycepin through DR3 pathway in the human colonic cancer cell HT-29. Food Chem. Toxicol..

[32] Lee H.H., Kim S.O., Kim G.-Y., Moon S.-K., Kim W.-J., Jeong Y.K., Yoo Y.H., Choi Y.H. (2014). Involvement of autophagy in cordycepin-induced apoptosis in human prostate carcinoma LNCaP cells. Environ. Toxicol. Pharmacol..

[33] Wang D., Zhang Y., Lu J., Wang Y., Wang J., Meng Q., Lee R.J., Teng L. (2016). Cordycepin, a Natural Antineoplastic Agent, Induces Apoptosis of Breast Cancer Cells via Caspase-dependent Pathways. Nat. Prod. Commun..

[34] Shao L.W., Huang L.H., Yan S., Jin J.D., Ren S.Y. (2016). Cordycepin induces apoptosis in human liver cancer HepG2 cells through extrinsic and intrinsic signaling pathways. Oncol. Lett..

[35] Zhou Y., Guo Z., Meng Q., Lu J., Wang N., Liu H., Liang Q., Quan Y., Wang D., Xie J. (2017). Cordycepin affects multiple apoptotic pathways to mediate hepatocellular carcinoma cell death. Anti-Cancer Agents Med. Chem..

[36] Lee H.H., Jeong J.-W., Lee J.H., Kim G.-Y., Cheong J., Jeong Y.K., Yoo Y.H., Choi Y.H. (2013). Cordycepin increases sensitivity of Hep3B human hepatocellular carcinoma cells to TRAIL-mediated apoptosis by inactivating the JNK signaling pathway. Oncol. Rep..

[37] Lee S.-J., Moon G.-S., Jung K.-H., Kim W.-J., Moon S.-K. (2010). c-Jun N-terminal kinase 1 is required for cordycepin-mediated induction of G2/M cell-cycle arrest via p21WAF1 expression in human colon cancer cells. Food Chem. Toxicol..

[38] Lee S.-J., Kim S.-K., Choi W.-S., Kim W.-J., Moon S.-K. (2009). Cordycepin causes p21WAF1-mediated G2/M cell-cycle arrest by regulating c-Jun N-terminal kinase activation in human bladder cancer cells. Arch. Biochem. Biophys..

[39] Yamamoto K., Shichiri H., Uda A., Yamashita K., Nishioka T., Kume M., Makimoto H., Nakagawa T., Hirano T., Hirai M. (2015). Apoptotic effects of the extracts of cordyceps militaris via Erk phosphorylation in a renal cell carcinoma cell line. Phytother. Res..

[40] Hwang J.-H., Joo J.C., Kim D.J., Jo E., Yoo H.-S., Lee K.-B., Park S.J., Jang I.-S. (2016). Cordycepin promotes apoptosis by modulating the ERK-JNK signaling pathway via DUSP5 in renal cancer cells. Am. J. Cancer Res..

[41] Joo J.C., Hwang J.H., Jo E., Kim Y.-R., Kim D.J., Lee K.-B., Park S.J., Jang I.-S. (2017). Cordycepin induces apoptosis by caveolin-1-mediated JNK regulation of Foxo3a in human lung adenocarcinoma. Oncotarget.

[42] Hwang J.H., Park S.J., Ko W.G., Kang S.-M., Lee D.B., Bang J., Park B.-J., Wee C.-B., Kim D.J., Jang I.-S. (2017). Cordycepin induces human lung cancer cell apoptosis by inhibiting nitric oxide mediated ERK/Slug signaling pathway. Am. J. Cancer Res..

[43] Tao X., Ning Y., Zhao X., Pan T. (2016). The effects of cordycepin on the cell proliferation, migration and apoptosis in human lung cancer cell lines A549 and NCI-H460. J. Pharm. Pharmacol..

[44] Zhang C., Zhong Q., Zhang X., Hu D., He X., Li Q., Feng T. (2015). Effects of Cordycepin on Proliferation, Apoptosis and NF-κB Signaling Pathway in A549 Cells. Zhong Yao Cai/Zhongyaocai/J. Chin. Med. Mater..

[45] Choi S., Lim M.-H., Kim K.M., Jeon B.H., Song W.O., Kim T.W. (2011). Cordycepin-induced apoptosis and autophagy in breast cancer cells are independent of the estrogen receptor. Toxicol. Appl. Pharmacol..

[46] Kim H., Naura A.S., Errami Y., Ju J., Boulares A.H. (2011). Cordycepin blocks lung injury-associated inflammation and promotes BRCA1-deficient breast cancer cell killing by effectively inhibiting PARP. Mol. Med..

[47] Wang Z., Wu X., Liang Y.-N., Wang L., Song Z.-X., Liu J.-L., Tang Z.-S. (2016). Cordycepin induces apoptosis and inhibits proliferation of human lung cancer cell line H1975 via Inhibiting the Phosphorylation of EGFR. Molecules.

[48] Noh E.-M., Jung S.H., Han J.-H., Chung E.-Y., Jung J.-Y., Kim B.-S., Lee S.-H., Lee Y.-R., Kim J.-S. (2010). Cordycepin inhibits TPA-induced matrix metalloproteinase-9 expression by suppressing the MAPK/AP-1 pathway in MCF-7 human breast cancer cells. Int. J. Mol. Med..

[49] Tian T., Song L., Zheng Q., Hu X., Yu R. (2014). Induction of apoptosis by *Cordyceps militaris* fraction in human chronic myeloid leukemia K562 cells involved with mitochondrial dysfunction. Pharm. Mag..

[50] Ko B.-S., Lu Y.-J., Yao W.-L., Liu T.-A., Tzean S.-S., Shen T.-L., Liou J.-Y. (2013). Cordycepin regulates GSK-3β/β-catenin signaling in human leukemia cells. PLoS ONE.

[51] Liao Y., Ling J., Zhang G., Liu F., Tao S., Han Z., Chen S., Chen Z., Le H. (2015). Cordycepin induces cell cycle arrest and apoptosis by inducing DNA damage and up-regulation of p53 in Leukemia cells. Cell Cycle.

[52] Baik J.-S., Mun S.-W., Kim K.-S., Park S.-J., Yoon H.-K., Kim D.-H., Park M.-K., Kim C.-H., Lee Y.-C. (2016). Apoptotic effects of cordycepin through the extrinsic pathway and p38 MAPK activation in human glioblastoma U87MG cells. J. Microbiol. Biotechnol..

[53] Li Y., Li R., Zhu S., Zhou R., Wang L., Du J., Wang Y., Zhou B., Mai L. (2015). Cordycepin induces apoptosis and autophagy in human neuroblastoma SK-N-SH and BE (2)-M17 cells. Oncol. Lett..

[54] Baik J.-S., Kwon H.-Y., Kim K.-S. (2012). Cordycepin induces apoptosis in human neuroblastoma SK-N-BE (2)-C and melanoma SK-MEL-2 cells. Indian J. Biochem. Biophys..

[55] Hsu P.-Y., Lin Y.-H., Yeh E.-L., Lo H.-C., Hsu T.-H., Su C.-C. (2017). Cordycepin and a preparation from Cordyceps militaris inhibit malignant transformation and proliferation by decreasing EGFR and IL-17RA signaling in a murine oral cancer model. Oncotarget.

[56] McIlwain D.R., Berger T., Mak T.W. (2013). Caspase functions in cell death and disease. Cold Spring Harbor Perspect. Biol..

[57] Cai J., Yang J., Jones D. (1998). Mitochondrial control of apoptosis: The role of cytochrome c. Biochim. Biophys. Acta.

[58] Gessi S., Merighi S., Sacchetto V., Simioni C., Borea P.A. (2011). Adenosine receptors and cancer. Biochim. Biophys. Acta.

[59] Antonioli L., Fornai M., Blandizzi C., Pacher P., Haskó G. (2018). Adenosine signalling and the immune system: When a lot could be too much. Immunol. Lett..

[60] Fredholm B.B., IJzerman A.P., Jacobson K.A., Klotz K.-N., Linden J. (2001). International Union of Pharmacology. XXV. Nomenclature and classification of adenosine receptors. Pharmacol. Rev..

[61] Ramkumar V., Stiles G., Beaven M., Ali H. (1993). The A3 adenosine receptor is the unique adenosine receptor which facilitates release of allergic mediators in mast cells. J. Biol. Chem..

[62] Ali H., Müller C., Daly J.W., Beaven M.A. (1991). Methylxanthines block antigen-induced responses in RBL-2H3 cells independently of adenosine receptors or cyclic AMP: Evidence for inhibition of antigen binding to IgE. J. Pharmacol. Exp. Ther..

[63] Waldo G.L., Ricks T.K., Hicks S.N., Cheever M.L., Kawano T., Tsuboi K., Wang X., Montell C., Kozasa T., Sondek J. (2010). Kinetic scaffolding mediated by a phospholipase C–β and Gq signaling complex. Science.

[64] Brown S.-A., Morgan F., Watras J., Loew L.M. (2008). Analysis of phosphatidylinositol-4, 5-bisphosphate signaling in cerebellar Purkinje spines. Biophys. J..

[65] Milella M., Kornblau S.M., Estrov Z., Carter B.Z., Lapillonne H., Harris D., Konopleva M., Zhao S., Estey E., Andreeff M. (2001). Therapeutic targeting of the MEK/MAPK signal transduction module in acute myeloid leukemia. J. Clin. Investig..

[66] Fresno J.V., Casado E., Cejas P., Belda-Iniesta C., González-Barón M. (2004). PI3K/Akt signalling pathway and cancer. Cancer Treat. Rev..

[67] Fishman P., Bar-Yehuda S., Liang B.T., Jacobson K.A. (2012). Pharmacological and therapeutic effects of A3 adenosine receptor agonists. Drug Discov. Today.

[68] Robinson D.R., Wu Y.-M., Lonigro R.J., Vats P., Cobain E., Everett J., Cao X., Rabban E., Kumar-Sinha C., Raymond V. (2017). Integrative clinical genomics of metastatic cancer. Nature.

[69] Ma Y.-H.V., Middleton K., You L., Sun Y. (2018). A review of microfluidic approaches for investigating cancer extravasation during metastasis. Microsyst. Nanoeng..

